# A New Perspective on Precision Medicine: The Power of Digital Organoids

**DOI:** 10.34133/bmr.0171

**Published:** 2025-03-24

**Authors:** Qian Yang, Mengmeng Li, Zian Xiao, Yekai Feng, Lanjie Lei, Shisheng Li

**Affiliations:** ^1^ Department of Otorhinolaryngology Head and Neck Surgery, The Second Xiangya Hospital, Central South University, Changsha 410011, Hunan, China.; ^2^ Key Laboratory of Artificial Organs and Computational Medicine in Zhejiang Province, Institute of Translational Medicine, Zhejiang Shuren University, Hangzhou 310015, Zhejiang, China.

## Abstract

Precision medicine is a personalized medical model based on the individual’s genome, phenotype, and lifestyle that provides tailored treatment plans for patients. In this context, tumor organoids, a 3-dimensional preclinical model based on patient-derived tumor cell self-organization, combined with digital analysis methods, such as high-throughput sequencing and image processing technology, can be used to analyze the genome, transcriptome, and cellular heterogeneity of tumors, so as to accurately track and assess the growth process, genetic characteristics, and drug responsiveness of tumor organoids, thereby facilitating the implementation of precision medicine. This interdisciplinary approach is expected to promote the innovation of cancer diagnosis and enhance personalized treatment. In this review, the characteristics and culture methods of tumor organoids are summarized, and the application of multi-omics, such as bioinformatics and artificial intelligence, and the digital methods of organoids in precision medicine research are discussed. Finally, this review explores the main causes and potential solutions for the bottleneck in the clinical translation of digital tumor organoids, proposes the prospects of multidisciplinary cooperation and clinical transformation to narrow the gap between laboratory and clinical settings, and provides references for research and development in this field.

## Introduction

Precision medicine, a highly customized medical model, is personalized medicine that uses advanced genomics technology to analyze the unique genetic characteristics and differences among individuals [1]. This process is not limited to the genetic level but also considers multidimensional information, such as the patient’s clinical condition, environmental factors, personal habits, and lifestyle, to ensure comprehensive and effective treatment interventions [2,3]. Precision medicine transforms traditional empirical medicine into data-driven precision decision-making, providing the most optimal treatment plan, which has become a hot topic of contemporary medical research. Results from clinical trials have shown that precision medicine can help improve treatment efficacy and patient prognosis through either genomic combined transcriptomic or combination therapies [4,5]. The implementation of precision medicine relies on the construction of preclinical models that are highly similar to the internal environment of the human body to simulate disease processes, evaluate the potential effects of treatment regimens, and predict clinical responses [6,7].

In this context, organoids have demonstrated great potential and application in precision medicine research owing to their ability to accurately simulate the structure and function of tissues and organs in vivo. Organoids retain the genetic information and microenvironmental characteristics of the original tissue cells and reproduce the occurrence and development mechanism of diseases to a certain extent. Organoids are ideal for exploring and optimizing treatment plans for specific patients and promoting the development of precision medicine [8]. Traditional tumor research methods, such as cell lines and animal models, can simulate tumor growth and development but often fail to completely replicate the real tumor conditions in patients [9]. However, tumor organoids can compensate for this deficiency and provide realistic tumor models [10]. Tumor organoids are usually 3-dimensional (3D) in vitro tumor models derived from patient-derived tumor cells, which contain multiple cell lineages, largely retaining the heterogeneity and complexity of the primary tumor and can simulate the biological behavior of the tumor, such as proliferation, invasion, and metastasis, in vitro [11,12].

Unlike 2-dimensional (2D) cell lines in which genetic drift during growth is difficult to avoid, the 3D growth environment provided by tumor organoids maintains the stability of the primary tumor mutation over a relatively long period [13]. Therefore, tumor organoids can facilitate in-depth studies of tumor pathogenesis, the exploration of effective therapeutic approaches, and drug efficacy evaluation [14,15]. When tumor organoids are combined with advanced technologies, the correlation between a patient’s genotype and drug response, as well as the prediction of biomarkers used for diagnosis, treatment, or determining prognosis, can be analyzed more accurately, providing a more rational reference for clinical decision-making [16].

Digital analysis methods have been progressively applied in organoid research. Notably, the use of high-throughput sequencing, image processing, bioinformatics, artificial intelligence (AI), and other technological tools has allowed for the comprehensive characterization and analysis of tumor organoids and aided researchers in elucidating the pathogenesis of tumors and identifying therapeutic targets [17–20]. The wide application of single-cell sequencing technologies has enabled the obtaining of rapid and accurate genomic information on tumor organoids, including gene mutations and chromosomal rearrangements [17]. This information is crucial for determining the genetic background and molecular mechanisms of tumors [21]. The rapid development of image processing technology and AI algorithms have also provided tools for the morphological analysis of tumor organoids [18,22,23]. Through automatic identification and quantitative analysis of high-resolution microscopic images of tumor organoids, researchers can objectively evaluate their growth, cellular heterogeneity, and interactions with the surrounding environment [24–27]. In addition, bioinformatic methods play an important role in integrating multi-omics data on tumor organoids. Integrating genomic, proteomic, metabolomic, and other multidimensional data provides a comprehensive understanding of the biological characteristics of tumors, facilitating the development of precise treatment strategies [28–30]. In addition to improving the accuracy and efficiency of research, these digital analysis methods also provide strong technical support for the application of tumor organoids in precision medicine. By observing the response of patient-derived organoids to different drugs, the efficacy of treatment can be accurately predicted, so as to tailor the most effective treatment for patients and avoid unnecessary drug side effects and treatment delays.

This review first summarizes the characteristics and culture methods of tumor organoids. The digital methods of organoids, such as bioinformatics, microscopic imaging technology, AI, and multi-omics, and the application of digital organoids in precision medicine are also discussed. Finally, this review explores the primary causes and potential solutions for the bottleneck in the clinical translation of digital tumor organoids, proposes the prospects of digitized organoids to narrow the gap between laboratory and clinical settings, and provides references for research and development in this field.

## Overview of Tumor Organoids

### Comparison with existing preclinical models

Currently, the primary preclinical models commonly used in oncology research are 2D cell culture lines, patient-derived tumor xenografts (PDXs), and patient-derived tumor organoids (PDOs) [31]. Among them, 2D cell culture lines are the oldest model, with mature and easy-to-use modeling methods, and have made important contributions to mechanistic studies of tumors, high-throughput drug screening, and large-scale histological studies. Haag et al. [32] found that histone H3 mutations induced specific results in different neural cells and that H3.3-K27M could drive tumorigenesis in neural stem cells but not in glial progenitor cells, a result that contributed to the understanding of diffuse endogenous pontine glioma development. However, the overall degree of overlap of aberrant genes between induced neural stem cells (iNSCs) inducing H3.3-K27M and PDX was low, which may be related to the survival pressure in vitro and in vivo. Cell lines are mostly derived from a single-cell species. The complexity of real tumor structures makes it difficult to reproduce the real state of the tumor in vivo and its interactions with the microenvironment in 2D cell lines, which may lead to their neglect of intra-/intertumor variations and biased prediction of treatment response [33]. In addition, the persistence of genetic drift in cell lines also adds uncertainty to drug-predicted responses [34]. The PDX model maintains tumor heterogeneity and tumor–stroma interactions by transplanting patient-derived tumor tissue into immunodeficient mice, which is closer to reality than subcutaneous xenografts [35]. However, the transplanted tumor microenvironment is susceptible to replacement by murine-derived cells during growth and passaging, resulting in tumor cells exhibiting different evolutionary trajectories between mouse models and patients [36,37]. In addition, the lack of a normal immune microenvironment because the tumor tissues are grown in immunodeficient mice may yield experimental results inconsistent with reality [38]. Coupled with the limitations of PDX, such as complex operation, low success rate, and lack of ease of gene editing, it has not been used in clinical settings.

Organoids are in vitro cultures of cells with 3D structures that can self-organize and exhibit structures and functions similar to those of their original or differentiated target organs [39]. Organoid-origin cells include embryonic stem cells (ESCs), induced pluripotent stem cells (iPSCs), adult stem cells (ASCs), and tumor cells [31]. ESCs and iPSCs exhibit multidirectional differentiation and almost infinite proliferation. Many organ models can be cultured through induced differentiation, which is important for studying human development [40,41]. ASC-derived organoids closely resemble the normal physiological state of the human body and are commonly used in infectious diseases [42,43], genetic diseases [44,45], disease modeling [46–48], regenerative medicine [49], and human body development [50,51]. ASCs can precisely differentiate into their source organs or tissues to replace and renew necrotic cells and maintain tissue stability in vivo, offering new possibilities for organ transplantation and personalized medicine [42]. Tumor organoids are usually derived from the patient’s tumor tissue or cells. This technique has the advantage of preserving the genetic characteristics and phenotype of PDX, genetic manipulation, and high-throughput drug screening of 2D cultured cell lines, compensating for some limitations of both (Table S1).

In summary, tumor organoid models, especially PDOs, combine the advantages of 2D cell culture and PDX models while overcoming their limitations, providing revolutionary new tools for tumor research, drug screening, and personalized treatment. PDOs can highly replicate the genetic and histological characteristics of patients’ tumors, preserve tumor heterogeneity, and show great potential in the application of precision medicine. Although further validation is still needed, they undoubtedly open up new avenues for the development of tumor treatment and precision medicine.

For example, cell lines cannot restore intratumor heterogeneity and histological features of patient tumors, and using animals in the passaging and culturing process of PDX is costly. PDO can recapitulate the genetics and histological features of patients’ tumors and better preserve inter- and intratumor heterogeneity, as well as patient’s responsiveness and resistance to treatment. PDO is used for drug screening and safety testing to tailor treatment regimens, improving survival rates and quality of life [43]. Thng et al. [52] matched advanced metastatic malignant tumor (metCRC) PDOs originating from primary and metastatic sites and obtained 3 matched pairs of PDOs. Epigenetic and drug combination analyses of these 3 pairs of PDOs predicted the drug sensitivity of the corresponding patients to single drugs or epigenetic-based combination therapies, assisting in developing patient treatment strategies. Notably, one of the PDO pairs showed different drug sensitivities despite having genetic similarity, which suggests the limitations of the genome in precision medicine applications. The application of PDO in precision medicine has reduced study time and facilitated real-time patient assessment and adjustment of treatment regimens, although the resulting predictions warrant validation in large cohorts and clinical trials. In addition, organoids of normal tissue origin can enable precancerous lesions to develop into tumors or cancers through gene-editing techniques, such as CRISPR-Cas9, and be used to model tumorigenesis and related omics studies.

### Culture methods

Culturing tumor organoids is initiated by obtaining patient tumor tissues or cells, usually derived from surgical resection, needle aspiration biopsy, or circulating tumor cells. To ensure the success rate of culture and the quality of the organoid, samples that are active, free of contamination, and rich in tumor cells should be selected. Immediately after obtaining the tissues, they are washed and minced to release the tumor cells from the tissues via enzymatic digestion or mechanical separation. After cell separation, a cell counting and viability assay is required to ensure that cell viability and purity meet the culture requirements. Culture medium is one of the key factors in tumor organoid culture. To mimic the tumor growth environment in vivo, the medium must provide the nutrients required for cell growth, such as amino acids, sugars, vitamins, and minerals. In addition, depending on the type of tumor, specific growth factors, hormones, and extracellular matrix (ECM) components must be added to mimic cell signaling, which, in turn, promotes the formation of tumor organoids [53].

The ECM component of traditional PDO culture protocols is selected to be Matrigel, an animal-derived and compositionally unknown ECM substitute that may affect cellular activity as well as reduce the reproducibility of PDO [54,55]. To avoid the potential adverse effects of Matrigel for PDO, researchers have developed various 3D scaffolds to suit different experimental needs. Haag et al. [32] indicated that scaffolds made of natural materials, such as collagen and gelatin, have good histocompatibility and can be perfect substitutes for Matrigel. The resulting colorectal organoids preserved the sensitivity of the patient’s tumor to drugs. However, the poor mechanical properties of these natural material scaffolds and the difficulty of independently controlling the biochemical and biophysical properties have limited their application [56]. In contrast, scaffolds made of synthetic materials, such as polylactic acid and polycaprolactone, have good controllability of physicochemical properties, which can adapt to the needs of the mechanical environment at different stages of the organoid culture process. Moreover, the small batch variation, combined with a medium with a well-defined composition, can largely improve PDO reproducibility and avoid the uncertainty added by unknown components to the experiment [57]. In addition, bioprinting technology controls the spatial distribution of cells and ECM with micron-level resolution, which can better mimic the in vivo tumor tissue structure; however, the cost of the current application is high [12]. Normal organoids mutated by gene editing are referred to as engineered tumor organoids, with CRISPR-Cas9 technology being the most widely used. Matano et al. [47] introduced mutations in the tumor suppressor genes APC, SMAD4, and TP53 and the oncogenes KRAS and/or PIK3CA into normal intestinal organoids using CRISPR-Cas9 genome editing technology. The mutated organoids were also provided with a culture environment containing inhibitors of Wnt, R-spondin, epidermal growth factor (EGF), and bone morphogenetic protein/transforming growth factor-β, which are essential for colorectal cancer (CRC) organoids of patient origin. Their results suggest that mutations in these genes can mimic adenomatogenesis but cannot induce the invasive behavior characteristic of malignant tumors.

In culturing tumor organoids, the growth, morphological changes, and metabolic activity of the cells must be regularly monitored via microscopic observation, flow cytometry, and metabolomics analysis. Multi-omics methods, such as high-throughput sequencing technology, can be used to assess the gene expression and protein composition of the tumor organoids to verify whether they successfully mimic tumor tissues in vivo. Through these monitoring and assessments, culture conditions and processing methods can be adjusted to ensure the quality and reliability of tumor organoids. Although PDO demonstrates excellent similarity in patient tumor genotype, histology, and treatment sensitivity, this technology remains in the preliminary stages of development and warrants improvements in the tumor microenvironment and vascular system [58]. In addition, the difficult access to patient tumor tissues/cells, lack of a standardized culture protocol for PDOs, and use of animal-derived substrates and sera make the created organoids less reproducible and controllable [59].

### Characteristics of tumor organoids

The characteristics of tumor organoids include histological and phenotypic/genotypic characteristics similar to those of the parent tumors. More accurate physiological modeling enables organoids to replicate the intra- and intertumor heterogeneity that exists in the body, which is conducive to the study of cancer development processes and discovery of new therapeutic targets [50]. Furthermore, the ability of organoids to capture subclonal populations may be more predictive of patient responses to treatment [60].

#### Similar histological characteristics to parental tumors

The prostate cancer organoid lines previously established by Gao et al. [61] and the tumor xenografts exhibit histopathological features of the parental tumor. Similar to the parental tumors, organoids established from bone marrow metastatic tumor cells showed intraductal growth and positive and negative immunohistochemical staining for pan-cytokeratin and androgen receptors, respectively.

Subsequent transplantation of bone marrow metastatic tumor organoids into immunodeficient mice showed the same histological and immunological features as in vitro organoids. Moreover, tumor organoids retained the histopathological features and corresponding functions of parental tumors in PDOs based on glioblastoma [62], primary liver cancer [48], esophageal adenocarcinoma [63], bladder cancer [64], and gastric cancer [65]. The growth and metastatic abilities of the parental tumor are retained following the transplantation of tumor organoids into animal models [48,62,66]. Therefore, PDOs are an effective model for in vitro studies on tumor development.

In contrast, cancer cell lines prepared in a culture medium exhibited markedly different histopathological features than those of the parental tumor, primarily due to the absence of ECM. The ECM provides cellular support, mediates cell polarity and intracellular signal transduction, and plays important roles in cell proliferation and differentiation [67]. Xu et al. [68] reported that lack of ECM inhibited the ability of 2D cultured mammary epithelial cells to functionally differentiate and express lactoproteins. Meanwhile, 3D-cultured mammary epithelial cells in an ECM environment form functional alveoli-like structures and establish tissue-specific carrier secretion, preserving the differentiation, function, and polarity of mammary epithelial cells [69].

In conclusion, prostate cancer organoids and tumor xenografts are highly similar to their parent tumors in histopathological characteristics and function, while traditional cancer cell lines lack ECM, which leads to differences in characteristics, highlighting the superiority and application potential of PDO in cancer research.

#### Similar phenotypic and genotypic characteristics to parental tumors

Different subpopulations of tumor cells exhibit intratumor heterogeneity (different performances in terms of oxygen uptake capacity, growth rate, and invasive capacity), an important feature for distinguishing tumor cells from normal cells [70]. Genotypic and phenotypic heterogeneity are the primary indicators for evaluating intratumor heterogeneity and classifying tumor subtypes [71,72]. Reflecting inter- and intratumor heterogeneity is the basis for an appropriate preclinical tumor model for cancer development and drug screening [73]. Currently, the 2D culture of tumor cells is the most widely used and well-established preclinical model, with significance in tumor research and drug screening. However, tumor cells are usually defective in DNA repair pathways and increase the instability of their genomes (i.e., higher than normal mutation rates) [74]. 2D-cultured tumor cells lack tumor microenvironment similar to that in vivo and lose the phenotypic and genetic characteristics of the parental tumor during long-term in vitro culture and passage [71]. Moreover, long-term culture may expose cells to frequent selection pressure, leading to the genetic drift of nonparental tumors, partly explaining the unsatisfactory efficacy and safety of most anticancer drugs in clinical trials [75].

PDOs can preserve the heterogeneity of parental tumors more effectively than tumor cell lines. The organoid model of breast cancer described by Sachs et al. [46] maintains consistency with parental tumors in terms of histopathology, hormone receptors, and HER2 and preserves the DNA copy number of parental tumors to a large extent after passaging. In addition, Weeber et al. [76] showed 90% somatic mutation similarity between PDOs and parental tumors in the same patient with CRC, and the correlation between the DNA copy number profile of PDOs and parental tumors was as high as 0.89.

The different mutated genes between PDOs and their parental tumors are not critical. Similarly, Gao et al. [61] showed the phenotypic diversity of prostate cancer by establishing organoid lines of prostate cancer with copy number signatures of the parental tumors.

A study using PDOs of liver metastases from multiple CRCs reported that the intermetastatic pharmacological heterogeneity of drug sensitivity among metastatic sites in patients with moderate disease did not significantly differ at the genomic level; however, the differences at the transcriptomic level were proportional to this heterogeneity [77]. Therefore, the representation of the phenotypic and genetic characteristics of the parental tumors by PDOs suggests the preservation of inter- and intratumor heterogeneity. Consequently, this will facilitate the prediction of new therapeutic targets and the exploration of drug resistance mechanisms of existing drugs.

In summary, intratumoral heterogeneity is a key feature that distinguishes tumor cells from normal cells, and traditional 2D culture models have limitations in preserving this heterogeneity. In contrast, PDOs can more effectively retain the heterogeneity and key genetic characteristics of the parent tumor, providing a more reliable model for cancer research and drug screening. The high fidelity of PDOs in terms of genotype and phenotype not only helps predict new therapeutic targets but also enables in-depth exploration of drug resistance mechanisms, thereby promoting the development of personalized medicine and precision treatment.

## Digital Technologies in PDO

### Multi-omics technology

**Fig. 1.**
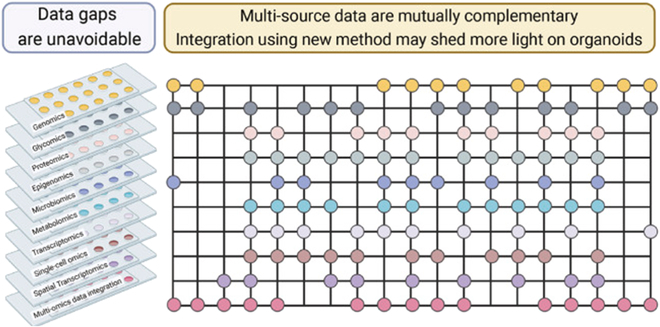
Multi-omics data in organoids [78]. Copyright 2023, Bai et al.

In the study of tumor organoids, multi-omics data include genomics, transcriptomics, proteomics, metabolomics, and other levels of data, which provide a comprehensive perspective for the study of tumor organoids (Fig. 1) [78]. Multi-omics technologies can analyze the gene expression profile of tumor organoids, determining and verifying whether PDO accurately summarizes the characteristics of the parent tumor [79,80]. Codrich et al. [81] used whole-exome sequencing to examine the timing of somatic variants according to patient characteristics and revealed adverse effects on CRC driver genes. Targeted NGS showed that PDO could accurately reflect the tumor characteristics of patients. A total of 5,125 differentially expressed transcripts have also been identified using RNA sequencing (RNA-seq), among which the phosphate and tension homology deleted on chromosome ten (PTEN) pathway attracted much attention and was verified by phosphoproteomics. The gene expression and genomic landscape of PDO revealed using whole-genome transcriptomic analysis and whole-exome sequencing analysis retain the characteristics of the parent tumor, and different tumor tissues and subtypes can still be distinguished under the same environment for a long time, which provides an excellent in vitro preclinical model for precision medicine, drug screening, and biomarker screening and identification [48]. Similarly, Papaccio et al. [82] generated 29 PDO lines from 22 patients with advanced CRC. Genomics and transcriptomics showed that their PDO could summarize the genotype and phenotypic characteristics of the parental tumors. The authors combined the sensitivity of PDO to the test drugs oxaliplatin or palbociclib, mass spectrometry, as well as the results of transcriptomics and quantitative proteomics, to perform integrated functional network analyses and identify different baseline protein and gene expression profiles. Oxaliplatin-resistant PDO exhibits enrichment toward the t-RNA aminoacylation process and shifts toward oxidative phosphorylation pathway dependence. In addition, palbociclib-sensitive PDO detected activation of MYC and enrichment of chaperonin T-complex protein ring complex.

The discovery of these protein and gene expression profiles may predict the treatment response or drug resistance in patients, providing ideas for precision medicine. Another study sequenced the genome, exome, and transcriptome of cells derived from patients with CRC, PDX, and PDO, respectively. PDO showed higher plasticity as a preclinical model for drug sensitivity screening. Multi-omics characterization of molecular features combined with drug susceptibility can identify new biomarkers of sensitivity to cetuximab (an EGFR inhibitor) [83]. In addition, multi-omics has been widely used in PDO for drug screening. Mun et al. [41] identified mitogen-activated protein kinase (MAPK) signal transduction heterogeneity in CRC organoids through targeted proteomics, and this tumor heterogeneity was associated with different responses to EGFR inhibitors. Multi-omics has also contributed to further high-throughput drug screening and drug testing in clinical development. Yan et al. [65] established a comprehensive and well-characterized organoid biobank of primary gastric cancer, which contained detailed whole-exome and transcriptome analyses. Drugs with good antitumor effects (napabucasin, abemaciclib, and the ATR inhibitor VE-822) were tested using this biobank. Therefore, multi-omics technology not only plays a unique role in determining the similarity between PDOs and patients’ tumors and reflecting the sensitivity of PDOs to drugs but also contributes to the accurate diagnosis and treatment of cancer and provides novel insights into the biological mechanisms of cancer.

**Fig. 2.**
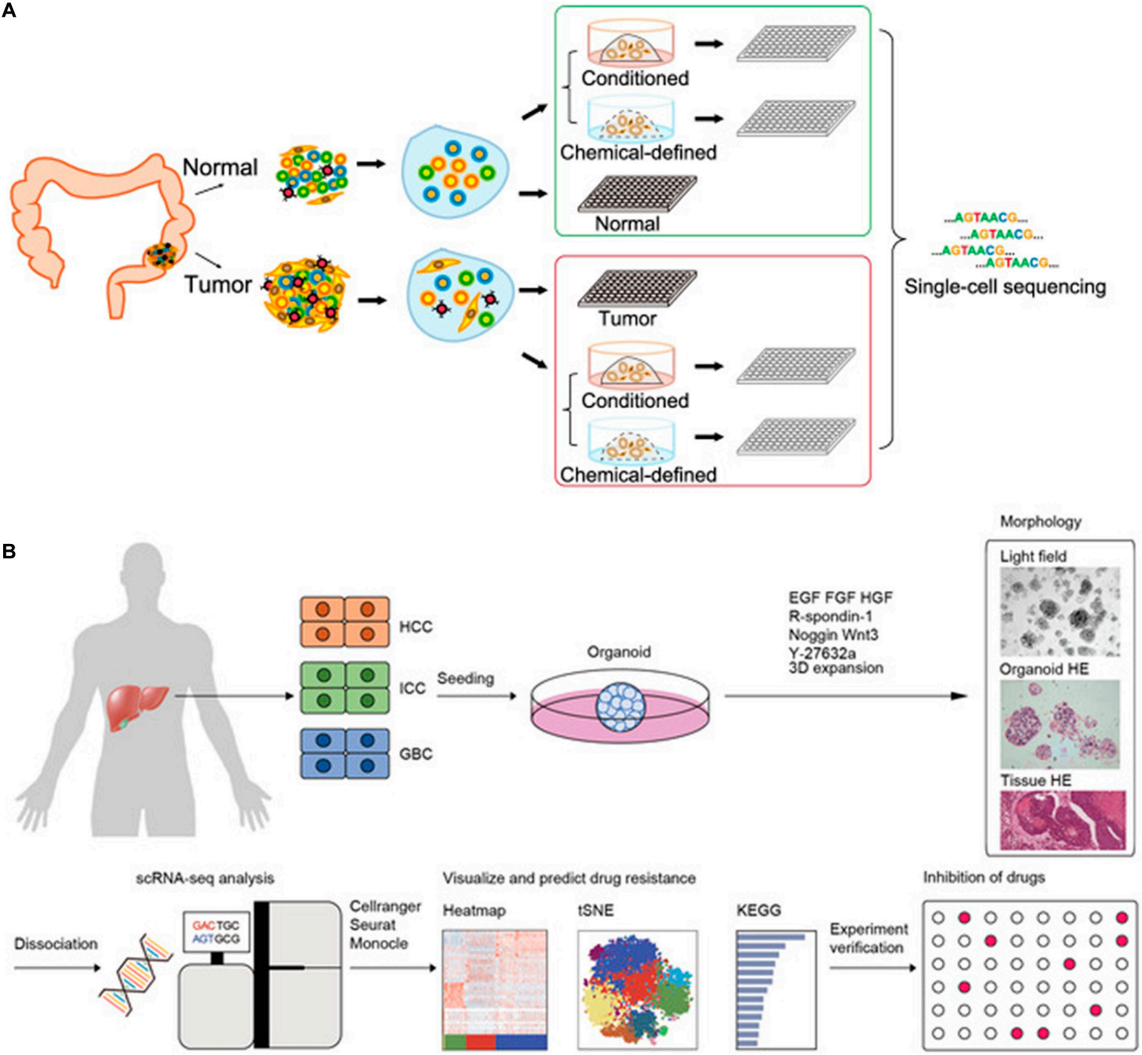
Application of single-cell sequencing in organoids. (A) Comprehensive analysis of tumor and paired adjacent normal tissue-derived organoids by single-cell RNA-seq [86]. Copyright 2022, Wang et al. (B) Flowchart of single-cell transcriptome analysis of intratumor heterogeneity and drug resistance in hepatobiliary tumor organoids [89]. Copyright 2021, Zhao et al.

Although whole-genome sequencing can provide a wealth of genetic information, it usually involves sequencing a mixed sample of a large number of cells, which cannot reveal the genomic variation of a single cell [84]. The emergence of single-cell sequencing technology (SCS) fills this gap. SCS can sequence the genome, transcriptome, and epigenome of individual cells, which further clarifies tumor heterogeneity and detailed genetic information (Fig. 2A) [85,86]. CRC organoids derived from multiple single cells show different characteristics at the genomic, epigenetic, transcriptomic, and functional level armies, and their responses to drugs differ markedly [87]. Kopper et al. [88] identified 56 ovarian cancer organoid lines by SCS and showed that these PDOs retained the intertumor and intratumor heterogeneity of the parental tumors. As PDOs are complex cell populations of different clones that differ at the genomic, transcriptomic, and epigenomic levels, SCS can capture these differences and reveal the characteristics and functions of different cell subsets (Fig. 2B) [89]. In addition, SCS can be used to study drug resistance mechanisms and metastasis processes of tumor cells, providing clues to overcome tumor drug resistance and develop new treatment methods [90]. Norkin et al. [91] developed a drug screening platform based on targeted organoid sequencing (Tornao-seq) that allows for a detailed assessment of cell phenotypes in CRC organoids and the detection of cell mixtures and their differentiation status in the intestinal system to isolate drugs that enrich differentiated cell phenotypes. The use of SCS can identify different cell types, cell states, and cell–cell interactions in tumor organoids, providing insights into the mechanisms underlying tumorigenesis, revealing the tumor microenvironment, and facilitating the development of effective therapeutic strategies [90,92].

Multi-omics data are usually derived from different experimental techniques and platforms and have the characteristics of high throughput, high dimension, and heterogeneity. Therefore, a series of preprocessing steps must be performed on the raw data prior to data integration. First, the genome and transcriptome data were pretreated with quality control, sequence alignment, and variation detection to ensure the accuracy and reliability of the data. Second, peak detection, alignment, material identification, and standardization of proteomics and metabolomics data were performed to extract useful information. These preprocessed multi-omics data are integrated for an integrated analysis to comprehensively and systematically describe the biological process. In this process, bioinformatics plays a key role by constructing network models to integrate omics data, reveal molecular interactions, and provide data mining and statistical analysis methods to identify tumor-related biomarkers, which provide new perspectives and potential diagnosis and treatment targets for cancer research. With the development of sequencing technology, the increasing amount of histological data from PDO has provided a rich information resource while making traditional data analysis methods seem overwhelming. Introducing AI can enhance the systematic analysis of experimental results.

In conclusion, multi-omics techniques are of great significance for studying tumor organoids. Integrating these techniques will clarify the complexity of tumor organoids, providing a more accurate and efficient method for the diagnosis, treatment, and development of personalized solutions for tumors.

### AI

AI is the discipline that researches and develops theories, methods, technologies, and application systems that simulate and extend human intelligence. It enables machines to perform tasks that require human intelligence, such as learning, reasoning, and problem-solving. AI technologies include machine learning (ML), deep learning, natural language processing, and computer vision, which create machine systems that can perceive, understand, learn, and solve problems [93]. ML is a core area of AI that uses data to train algorithms to recognize patterns and make predictions. With constant input of data and feedback, ML models can optimize their performance for more complex tasks [78]. Tumor organoids, as an emerging in vitro tumor model, provide a new application scenario for AI. PDO produces a large number of multidimensional raw data during the culture process, including cell imaging, omics map, phenotypic reading, and time series. Manual processing and analysis are time-consuming, laborious, and easy-to-miss data, while AI can provide more comprehensive, faster, and more effective analysis [94].

**Fig. 3.**
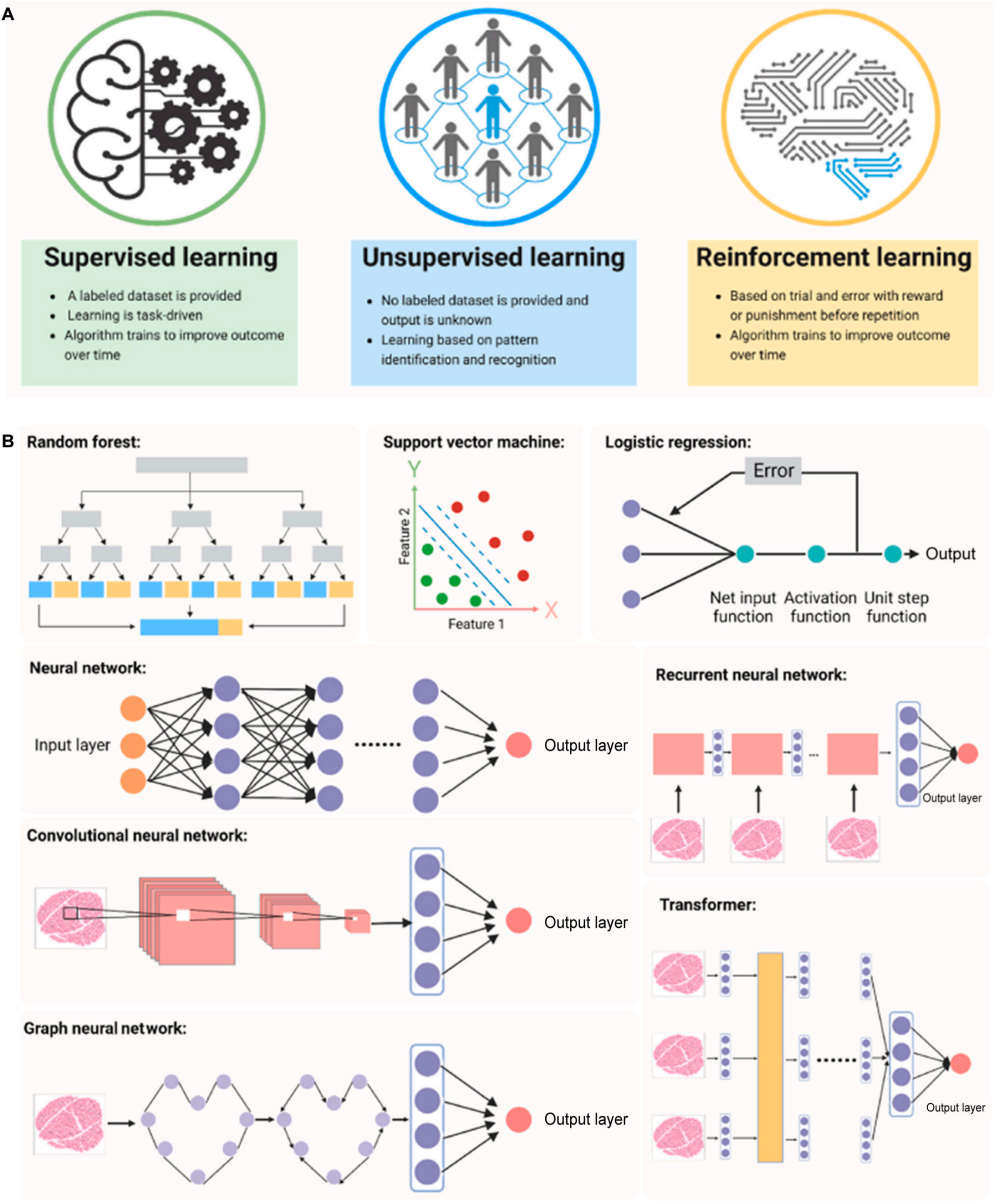
Common machine learning (A) types and (B) algorithms [78]. Copyright 2023, Bai et al.

The main ML types include supervised learning, unsupervised learning, and reinforcement learning [95]. Supervised learning is the process of learning a model from labeled training data, where computers make predictions and classifications by fitting mapping relationships between input features and output labels. The higher the accuracy of the classification labels and the more representative the sample, the more accurate the learned model. Typical supervised learning algorithms include regression and classification [96]. Unsupervised learning is the process of identifying patterns and structures in unlabeled training data. Common unsupervised learning algorithms include clustering, dimensionality reduction, random forest, and principal component analysis. Unlike supervised learning, unsupervised learning does not require training samples and manually labeled data, which facilitates compression of data storage, reduces the amount of computation, improves the speed of the algorithm, and avoids classification errors attributed to positive and negative sample bias. This learning method aims to provide meaning to the data by mining the underlying patterns in the data [97]. However, reinforcement learning is based on reward signals, in which an intelligent body (agent) takes a series of actions by interacting with the environment and receives reward or punishment signals from the environment. Through continuous trial and error and optimization, the agent learns to take the best action in a given environment to achieve its goal. In this learning model, input data provide feedback to the model and is only a way to determine whether the model is right or wrong. Common algorithms include Q-Learning and temporal difference learning (Fig. 3) [78,98,99]. ML empowers AI to learn, adapt, and process data by providing it with tools and methods, and improves its performance.

**Fig. 4.**
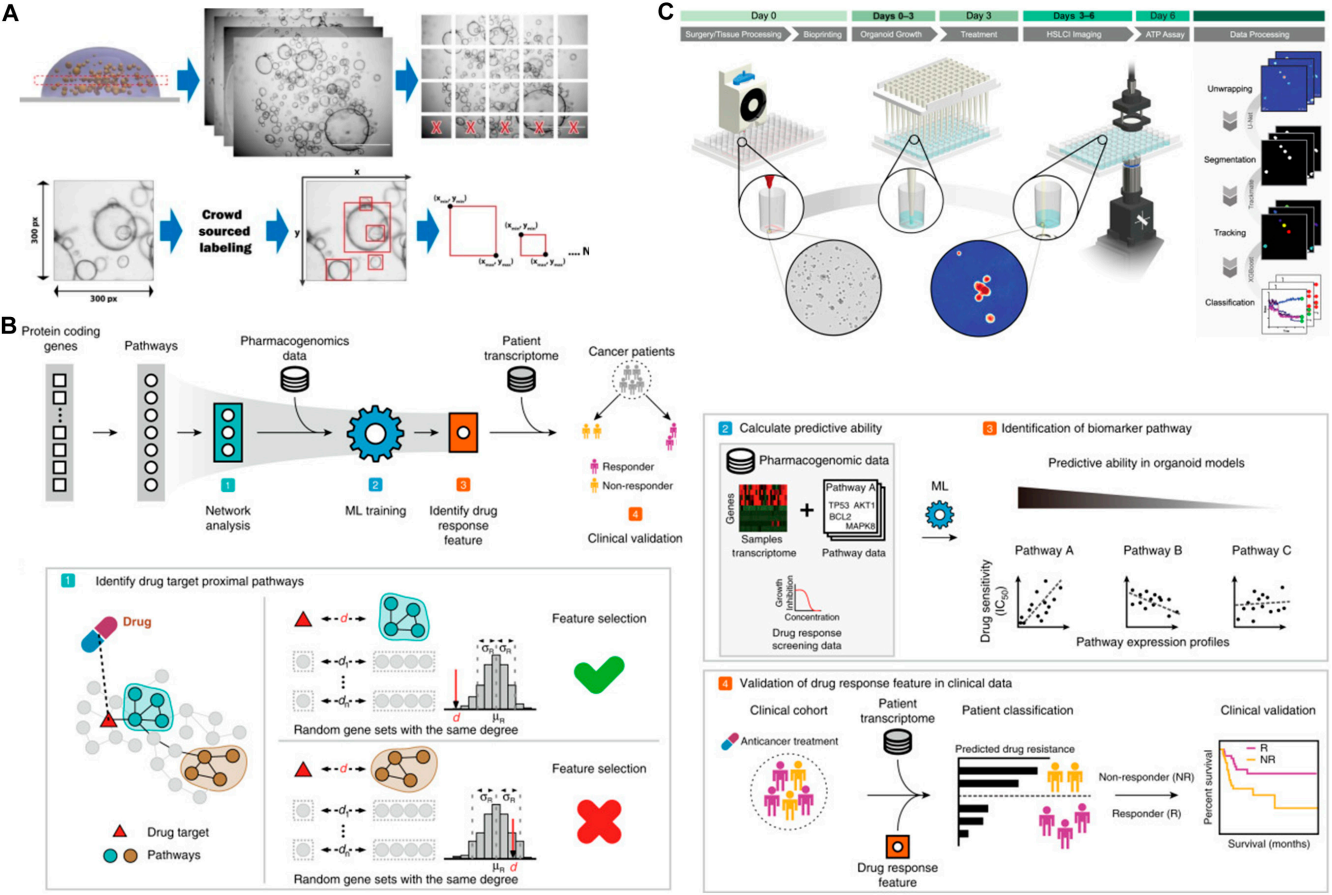
Application of AI/machine learning in organoids. (A) OrgaQuant automatically locates and quantifies the size distribution of human intestinal organoids in light field images [109]. Copyright 2019, Kassis et al. (B) Colorectal and bladder organoid models were analyzed by machine learning to predict patients’ anticancer drug efficacy [103]. Copyright 2020, Kong et al. (C) High-speed live-cell interferometry imaging organoids at single-organoid resolution [101]. Copyright 2023, Tebon et al.

At present, the application of ML in PDO focuses on analyzing organoid images. During PDO construction and characterization, optical microscopy image data generated from cellular experiments are crucial in assessing organoid function. These data reveal important information regarding organoid function by analyzing cell counts, morphology, and behavior. Changes in cellular characteristics reflect differentiation and response to environmental stimuli. However, traditional analysis methods are time-consuming and subjective, making it difficult to accurately quantify complex features. ML can improve the speed and accuracy of analysis by training algorithms to identify and quantify cellular features, significantly increasing the efficiency of organoid assessment and reducing manual work. ML uses image processing and pattern recognition algorithms to identify and classify images of tumor organoids. Tumor cells can be identified and segmented; their morphology, size, and density can be quantitatively assessed; and their biological properties can be inferred (Fig. 4C) [100,101]. Organoid features reveal their function and maturity, e.g., size and shape reflect the growth and development of the organoid, and the internal structure shows the organization and differentiation within organoids. Therefore, accurate analysis of organoid images is essential for functional assessment. This analysis involves a large amount of data acquired through 3D imaging or confocal microscopy, whereas traditional manual analysis methods are time-consuming, biased, and difficult to fully capture the structural complexity. Stüve et al. [102] have developed a high-throughput imaging analysis platform by integrating automated imaging techniques, image processing tools (e.g., greyscale conversion, contrast enhancement, membrane detection, and structure separation), and ML. The platform can identify and classify organoids cocultured with immune cells and perform high-throughput analyses of parameters, such as organoid number, size, and shape in the process. Based on this platform, the authors clarified that growth factors can adversely affect organoid growth and development. The significance of this study was that the proposed high-throughput imaging solution offers new possibilities for further analyzing and detecting organoids in coculture systems and has significant potential for refining preclinical models for precision medicine. In addition, ML can realize automatic quantification and tracking of organoid changes to track organoid growth and morphological changes, avoiding the complexity and error prone to manual screening [24]. Bian et al. [24] proposed a deep neural network that can effectively detect and dynamically track organoids. The high-throughput sequential images of all organoids are first processed frame by frame, then the similarity of organoids in adjacent frames is calculated, and the organoids on adjacent frames are matched in pairs, which finally form a high-throughput organoid image dataset, and the dataset is annotated in detail by an expert. Their solution enables the tracking and monitoring of organoid culture throughout its life cycle, which reduces labor costs and increases the success rate of organoid culture.

The construction and application of organoids require the assessment of multiple cellular interactions from multiple dimensions, which generates massive and complex multi-omics data and poses a great challenge to traditional analytical methods. ML can be used for data analysis and interpretation of multi-omics, such as genome and proteome of tumor organoids, and evaluate toxicity and simulate disease heterogeneity in drug screening (Fig. 4B) [103]. Chen et al. [104] revealed the transcriptome landscape of CRC organoids via single-cell sequencing and unbiased clustering of PDOs using the t-SNE algorithm (an ML algorithm for nonlinear dimension reduction based on probability distribution). The results showed a high diversity of tumor heterogeneity and subtypes retained in PDOs. Further, single-cell sequencing of PDO treated with oxaliplatin, analyzed using the t-SNE algorithm and compared with the untreated results, delineated 3 distinct subtypes of cell populations: drug-insensitive, drug-sensitive, and drug-hypersensitive groups, where the drug significantly interfered with the subtypes of PDO. This study demonstrates the promise of ML in omics data analysis and drug screening.

Deep learning is a branch of ML inspired by the structure and function of human neural networks. By establishing a multilevel complex network structure, deep learning can extract and learn high-level features in the input data to realize the recognition and prediction of complex patterns [105]. Compared with ML, deep learning can more flexibly process unstructured data types such as images, text, and speech [106]. Since organoids are 3D culture models, the dense number, diverse types, and tight connections of cells make cell segmentation and phenotype annotation of organoid images particularly difficult [25]. In the morphological analysis of tumor organoids, deep learning technology can identify and extract key features in images, providing a convenient method for quantitative analysis and comparison [107]. Convolutional neural network (CNN) is a typical application of deep learning in image processing that learns the nonlinear mapping relationship between the original image and the target feature by training on a large number of labeled images [108]. In organoid image analysis, CNN can locate and quantify organoids based on bright-field images of organoids in a 3D culture environment to determine the morphology, distribution, and growth characteristics of organoids, which greatly improves the analysis efficiency and accuracy (Fig. 4A) [109,110]. Matthews et al. [111] constructed the platform OrganoID based on the CNN network of U-NET, which identified, labeled, and tracked individual organoids pixel by pixel in bright-field and phase-contrast microscopy experiments.

Individual count and size accuracy were 95% and 97%, respectively. Deep learning can also be used to simulate the growth process of tumor organoids and optimize culture parameters and conditions. Constructing deep learning models to simulate organoids’ growth dynamics and cell-to-cell interactions can predict organoids’ growth under different culture conditions and provide guidance for experimental design, reducing costs and time and improving research efficiency [78]. Deep learning enables the analysis and mining of histological data from multiple scales and modalities. It integrates and compares them at the molecular, cellular, spatial structural, and organ levels, simplifying manual analyses’ workload and enhancing information mining capabilities.

ML and deep learning have demonstrated excellent performance, although further research remains warranted to address existing problems that limit further application. First, ML and deep learning have high requirements for the quantity and quality of data, while the nonstandardization and batch differences in the training process of PDO, as well as the inconsistency of data sources and formats, pose challenges to the accuracy, repeatability, and generalization of the model. Moreover, the existing studies do not have enough interpretation of AI models and only use performance indicators, such as accuracy or receiver operating characteristic. The high requirements of computing resources make it difficult to control the application cost, and the ethical and legal issues attributed to using AI limit its application. Despite the above shortcomings, AI has broad application prospects in organoid culture, characterization, omics data mining, and high-throughput image analysis.

### Microscopic imaging technique

Microscopic imaging is the basis for observing and analyzing the morphology of tumor organoids. Although traditional microscopic imaging methods, such as light microscopy and electron microscopy, can visualize images of tumor cells and tissue structures, these methods have limitations in resolution, contrast, and imaging depth [65].

In recent years, with the development of fluorescence microscopy, confocal microscopy, and super-resolution microscopy, high-resolution and multilevel images of tumor organoids can be obtained. These techniques not only greatly improve the sharpness and contrast of images but also enable the labeling and tracking of specific cellular structures and molecules, facilitating the in-depth study of the morphological characteristics of tumor organoids [112,113]. By combining different dyes and markers, these methods can implement specific cell types or molecules of the 3D positioning and visualization. Dekkers et al. [113] created a homemade fructose-glycerol hyaluronan that enables antibodies to penetrate the cells of the organoids and bind to intracellular proteins. The choice of single-photon confocal, multiphoton, and light sheet fluorescence microscopy (LSFM) for photography allowed normal organoids containing fluorescent reporter genes or immunolabeling and breast tumor organoids to be captured in full 3D. The protocol can achieve quantification of markers in individual cells of a tumor and significantly reduces the time for image acquisition and analysis. This can help to reveal the internal structure and spatial relationship of tumor organoids and provide an important reference for drug screening and treatment strategies.

In addition, in vivo imaging technology has been used to study organoids. This technique allows researchers to observe the growth and dynamic changes of tumor organoids in real time without destroying the sample. By combining different fluorescent markers and imaging techniques, intravital microscopy can reveal key biological processes, such as tumor–cell interaction, angiogenesis, and drug response. Toshimitsu et al. [114] used green fluorescent protein to label organoids and automatic imaging and conducted measurements using a high-content analyzer during organoid culture to calculate organoid growth based on fluorescence area.

Regarding quantitative analysis, extraction methods based on image processing can automatically measure and count key morphological parameters in tumor organoids, such as cell size, shape, and nuclear–cytoplasmic ratio [107,115]. These indicators are closely related to the tumor malignant degree and invasive ability to evaluate treatment effects and predict disease progression. In addition, optical metabolic imaging (OMI), a label-less assessment technique, uses autofluorescence of the metabolic coenzymes NAD(P)H and FAD to quantify the treatment response of all cells in an organoid. The noninvasive characteristics make it possible to detect the metabolic activity of organoids at any time point [116]. These quantitative analysis methods, combined with deep learning technology, can realize the automatic processing and analysis of large-scale imaging data, which provides strong support for high-throughput screening and precision medicine.

Modern microscopic imaging techniques provide high-resolution, multilevel image support for the study of tumor organoids and realize the precise positioning and tracking of cellular structures and molecules. Combined with specific staining and labeling techniques, these methods not only reveal the internal structure and spatial relationships of tumor organoids, but also facilitate drug screening and the development of therapeutic strategies. At the same time, the integration of quantitative analysis methods and deep learning technology realizes the automatic processing and analysis of large-scale image data, provides strong support for high-throughput screening and precision medicine, and promotes the in-depth development of tumor research.

## Integration of Digital Tumor Organoids and Precision Medicine

### Construction of individualized tumor models

Individualized tumor model construction facilitates accurate tumor diagnosis. Through an in-depth study of these models, clinicians can better understand the characteristics of a tumor, including its growth rate, drug sensitivity, and potential risk of metastasis. ML, multi-omics, and microscopic image technology play a key role in the construction and characterization of tumor organoid models and work synergistically with each other. Micrographics can provide a large number of high-resolution images of tumor organoids, and ML can identify and segment organoids in these images to label and quantify the size and location of the organoids. ML can track the organoids to clarify the growth process and growth rate [109,117]. Gunnarsson et al. [27] combined high-throughput imaging deep learning with mathematical modeling to create an innovative workflow for analyzing PDO growth kinetics, applicable to colon cancer organoids. They found that the Gompertz model was effective in describing organoid growth and revealed significant heterogeneity in growth kinetics within patients, with initial exponential growth rates being lognormal distributed. In addition, organoid growth rates and the duration of single-cell dormancy varied among patients. These findings contribute to the understanding of the essential characteristics of PDO and provide a research basis for predicting treatment response and drug resistance. The premise of fully and correctly extracting image features is high-quality images. When the quality of microscopic images is not high enough under the influence of objective factors, deep learning can reconstruct or correct them [118]. In addition, factors such as 3D scaffolds and culture conditions play an important role in organoid culture and significantly influence organoid growth and development. Different organoids have different requirements for the physical and chemical properties of 3D scaffolds. ML helps to design or optimize scaffold development by analyzing large datasets to determine how different components affect the final performance of the scaffold and can potentially enable customized scaffolds to enhance organoid research [119]. The spatial structure affects the attachment, proliferation, differentiation, and migration of cells on scaffolds. By analyzing imaging data from 3D scaffolds, ML can identify patterns of scaffold alignment and predict their impact on cell behavior, facilitating the optimization of scaffold structures for organoid growth. This technology provides insights into the effects of scaffold spatial structure on cellular behavior, potentially revolutionizing organoid research and providing tools to optimize organoid construction strategies.

**Fig. 5.**
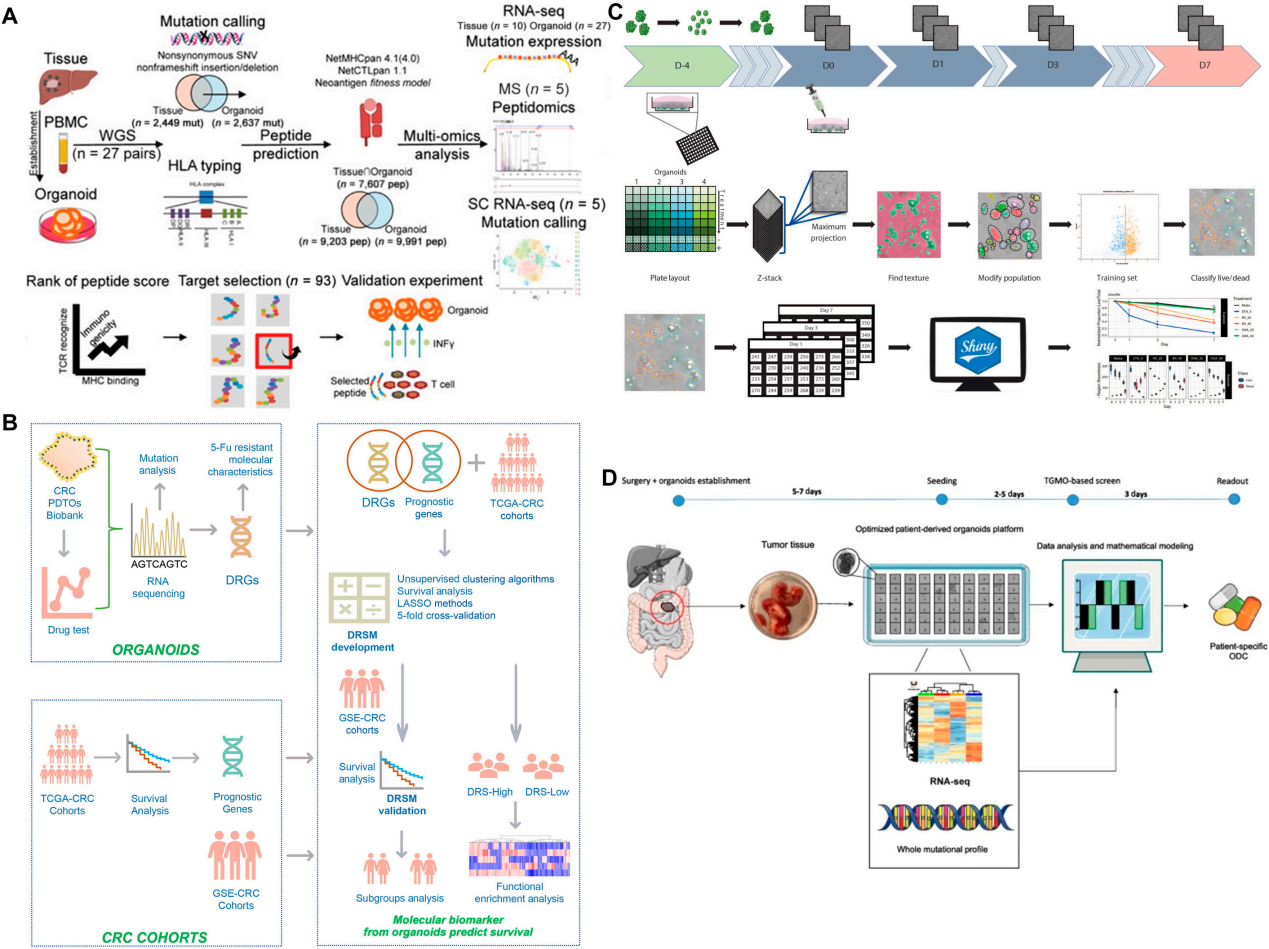
The application of digital tumor organoids in precision medicine. (A) Multi-omics analysis of hepatobiliary tumor organoids was used to identify neoantigen peptides for individual immunotherapy [120]. Copyright 2022, Wang et al. (B) Flowchart for the development of molecular biomarkers of drug resistance to predict survival in CRC patients based on patient-derived tumor organoids [122]. Copyright 2022, Chen et al. (C) Integrated imaging, computer vision, and machine learning to track and analyze the dynamic response of organoids to drugs [126]. Copyright 2021, Spiller et al. (D) Design tailored synergistic multidrug combinations for patients through multi-omics characterization and mathematical model prediction of patient-derived organoids [128]. Copyright 2023, Ramzy et al.

Tumor organoids must be characterized and evaluated in multidimensions following construction to determine whether PDO retains the characteristics of the parent tumor, which will determine its accuracy as a research model. Multi-omics technology from multiple molecular levels explores the biological characteristics of tumor organoid collection, including genome, transcriptomes, and proteome of comprehensive data, through bioinformatics analysis, revealing tumors at the molecular level with the parent class organs of similarity (Fig. 5A) [28,120]. Peng et al. [121], using ML algorithms, developed the CancerCellNet calculation tool, which can effectively block the different experimental platforms and the differences between species. This enabled the use of transcriptomic data to accurately and quantitatively assess the similarity of the cancer model to 22 naturally occurring tumor types and their 36 subtypes. The author applied the developed tool to determine similarities between tumor organoids, cell lines, patient-derived xenografts, and genetically engineered mouse models and patient tumors. The results showed that the tumor organoids and the genetically engineered mouse models exhibited higher transcriptional fidelity than the other 2 models. Furthermore, the molecular subtypes of tumors were closely related to the prognosis of patients. Identifying and classifying organoid subtypes is also an important step in determining the degree of intratumor heterogeneity reduction and modeling the real condition of the patient’s tumor. Okamoto et al. [23] classified CRC organoids into 6 types based on morphology and used this as a classification training dataset. The authors used these data to train 2 classifiers, a support vector machine and DensNet201. The training results showed that the accuracy of these classifiers was comparable to that of human classification. After completing the training of the whole module, the system can identify and classify CRC organoids in the image with full automation and high accuracy, which provides important support for the subsequent analysis of gene differential expression in organoids with different shapes. Thus, ML confirms that PDO can faithfully reflect the characteristics of the patient’s tumor and accurately performs the analysis by avoiding the interference of redundant information as much as possible.

The construction of individualized tumor models combines ML, multi-omics, and microscopic imaging technology, which provides a powerful tool for accurate tumor diagnosis. These techniques not only reveal tumor characteristics in depth, such as growth kinetics and drug sensitivity, but also optimize organoid culture conditions, ensuring a high degree of similarity between the model and the parental tumor. Through multidimensional characterization and evaluation, individualized tumor models have shown great potential in predicting treatment response, understanding tumor heterogeneity, and developing new therapies, which opens up new avenues for precision medicine and cancer research.

### Prognosis evaluation

The evaluation of prognosis is important for tumor treatment. By understanding the patient’s condition and prognosis, timely interventions can be employed to prolong survival and improve patients’ quality of life. Broutier et al. [48] first conducted a systematic analysis of the transcriptome of well-characterized healthy liver organoids and primary liver cancer organoids and identified 30 potential biomarkers, 19 of which have been reported as markers of primary liver cancer, while the remaining 11 have not been reported to be associated with it. Thirteen of the 19 genes that have been reported are strongly associated with poor prognosis. Further exploration of these 30 genes in a cohort of primary liver cancer patients and healthy individuals revealed 11 new genes, 4 of which were associated with poor prognosis: *C19ORF48*, *UBE2S*, *DTYMK*, and *C1QBP*. Chen et al. [122] used transcriptome sequencing to identify differentially expressed genes related to 5-fluorouracil (5-FU) resistance in patient-derived CRC organoids and, on this basis, combined it with LASSO regression and cross-validation to establish a 5-molecule gene signature and resistance scoring model. Validation showed that the model could predict the survival rate of CRC patients, and the drug resistance score was an independent prognostic factor for overall survival (Fig. 5B). These findings show that tumor organoids have great potential in identifying possible prognostic biomarkers and evaluating patient prognosis, and the combination of digital technologies and methods strengthens the advantages of tumor organoids as preclinical models.

### Treatment sensitivity prediction

Significant differences were observed in patients’ responses to different drugs in cancer treatment. Therefore, understanding patients’ sensitivity to specific drugs is crucial for developing precise treatment strategies. By constructing individualized tumor organoids, various candidate drugs can be tested and evaluated for tumor inhibition in vitro, and the accuracy and efficiency of their predictions can be improved after digital methods. Pasch et al. [123] used light field images and OMI to evaluate the differential response of PDO to chemotherapy and radiotherapy and predict the response of tumor organoids to 5FU, oxaliplatin, and combination therapy in patients with refractory metastatic CRC. The authors found poor responses to 5FU alone. Subsequent clinical retreatment also reflected the same nonresponse to 5FU. Similarly, Kong et al. [103] used ridge regression, linear regression, and support vector regression to integrate the pharmacogenomic data of colorectal and bladder cancer organoids to construct a drug response prediction model, which successfully predicted the efficacy of 114 CRC patients treated with 5-FU and 77 bladder cancer patients treated with cisplatin; the survival rate of predicted responders was significantly higher than that of nonresponders. This approach effectively identified biomarkers of drug response, improved therapeutic efficacy, and narrowed the gap between organoid research and clinical application (Fig. 4B). After confirming the correlation between the radiation response of rectal cancer organoids and the actual radiotherapy results, Park et al. [124] used the random forest method of ML to establish a radiotherapy outcome prediction model. The prediction accuracy reached 81.5% and 92.1%, respectively. The authors predicted or screened the most effective drugs or treatments through tumor organoid models, avoiding unnecessary adverse effects and healthcare costs. The above studies fully reflect the accurate prediction of drug sensitivity in tumors by digital PDO and provide a tool for developing precision medicine. In addition, PDO can be used for drug sensitivity testing and provide insights into existing or new therapeutic strategies. Yan et al. [13] detected for the first time an R-spondin fusion protein in early-onset CRC organoids, which had been previously reported only in cell lines, a finding beneficial for the clinical application of Wnt secretion inhibitors or anti-RSPO mAb. Bolhaqueiro et al. [125] performed live-cell imaging and single-cell karyotype sequencing of CRC organoids and found that chromosomal instability (CIN) is widespread in CRC and can coexist with microsatellite instability. The results suggest that high levels of PDO in CIN are sensitive to drugs that increase the frequency of chromosome segregation errors. Würth et al. [19] developed a long-term CTC-derived organoid model from patients with metastatic breast cancer. Multi-omics analysis and xenograft modeling revealed the central role of neuregulin 1 (NRG1) and ERBB2 receptor tyrosine kinase 3 (ERBB3/HER3) signaling in CTC survival, growth, and dissemination. In addition, a genome-wide CRISPR activation screen revealed that fibroblast growth factor receptor 1 (FGFR1) signaling could compensate NRG1–HER3 axis function to rescue NRG1-deficient circulating tumor cells (CTCs). Interestingly, NRG1–HER3 activation confers resistance to FGFR1 inhibition in CTCs, whereas combined blockade of these 2 signaling pathways effectively inhibits CTC growth. These findings reveal the molecular basis of cancer cell plasticity and suggest new strategies for clinical treatment. On the other hand, due to the faithful reflection of the characteristics of the patient’s tumor, the model can identify and verify the specific vulnerability of the patient and then become an innovative tool for precision medicine.

During sensitive drug screening, the choice of methods to evaluate the therapeutic effect is particularly important. Conventional viability assays (e.g., adenosine triphosphate levels) or fluorescent dyes are invasive and do not reflect intratumor heterogeneity well. The combination of microscopic imaging technology and AI enables real-time, dynamic, and noninvasive monitoring of organoid response to drugs. Spiller et al. [126] acquired light field images of 178 CRC organoids by high-content microscopy and used these data to train an ML linear model that achieved 78% and 61% agreement with expert and DRAQ7 staining on the test set, respectively. In addition, the authors used the model to successfully track the dynamic response to the drug in 3 patients (Fig. 5C). Larsen et al. [127] constructed a treatment analysis model based on a regularized conditional adversarial network, which was trained to predict fluorescence and viability readings using light field images. Not only did it enable the monitoring of drug responses by predicting fluorescent living dye staining phenotypes by optical microscopy, but it also reduced the cost and time of screening. This model offers new possibilities for personalized in vitro drug testing with higher reproducibility and less biomass input than metabolism-based assays.

In summary, the digital tumor organoid model combined with multi-omics analysis and AI technology has shown significant advantages in accurately predicting drug sensitivity, optimizing treatment regimens, revealing drug mechanisms, and discovering new therapeutic targets, which provides powerful tools and strategies for the development of personalized cancer treatment and precision medicine.

### Optimizing the combination therapy

Combination therapy, using multiple drugs simultaneously to enhance the therapeutic effects and reduce the risk of drug resistance, is a common strategy in cancer treatment. However, predicting interactions and synergistic effects of different drugs remains challenging, rendering the development of combination treatment regimens difficult. Digital tumor organoids play an important role in optimizing combination therapies, facilitating the testing of various drug combinations in organoids, and observing their effects on tumor inhibition. Ramzy et al. [128] combined whole exome and transcriptome sequencing with second-order linear regression and adaptive LASSO to discover optimized drug combinations (ODCs) in CRC. In tumor organoids from patients with stage IV CRC, the ODCs of regorafenib (1 mM), verofinil (11 mM), palbociclib (1 mM), and lapatinib (0.5 mM) markedly inhibited tumor–cell viability and were superior to clinical doses of first-line agents (Fig. 5D).

Accordingly, the drug combination with the best synergistic effect could be screened through comparative analysis, providing a scientific basis for formulating a combined treatment. Ji et al. [79] analyzed all possible combinations of 76 drugs based on proteogenomic data and screened the enriched pathways for each combination, from which the effectiveness and synergistic effects of drug combinations were clarified. This demonstrates that tumor organoids can be used to study the interaction mechanisms between drugs, reveal the molecular basis of drug synergy, and provide ideas for developing new combination therapies [79].

In summary, digital tumor organoids combined with multi-omics sequencing and data analysis technology effectively optimize the combination therapy of tumors. By screening the best drug combination and revealing the interaction mechanism, it provides important basis and ideas for scientific formulation of combination therapy strategies and development of new therapies.

## Challenges and Possible Solutions

### Challenges in clinical translation

#### Data analysis and interpretation

Multi-omics data cover multiple levels of tumors, but there are various types and sources of data, and there is heterogeneity, which makes data analysis more complicated. For example, different sequencing platforms and experimental methods may lead to differences in data that require standardization and adjustment. Secondly, integrating data from different omics platforms into one platform for effective analysis and interpretation is a great challenge. Deep learning models have achieved great success in data analysis, but their “black box” nature limits their interpretability and makes it difficult to understand the biological mechanisms behind the model predictions [129].

#### Model standardization and complexity

The culture conditions of organoids (such as medium composition, temperature, humidity, gas environment, etc.) are crucial for the development and characteristics of organoids, but small changes in culture conditions may lead to differences in organoids and affect the reliability and reproducibility of the model. Second, the cellular origin (such as patient origin or stem cell origin) and genetic background (such as gene mutations and epigenetic modifications) of organoids have important effects on organoid properties. The diversity of cell origin and genetic background needs to be considered and the corresponding classification and criteria need to be established.

Existing organoid models usually only contain tumor cells and lack other tumor microenvironment cells, such as immune cells, vascular endothelial cells, and stromal cells, which limits the complexity and predictive ability of organoid models [130]. Although tumor organoids have made progress in simulating tumor microenvironment, there is still a gap compared with the complex tumor environment in the human body [122]. In addition, the composition of media is expensive and difficult to replicate, and the use of animal serum may introduce heterogeneous components, affecting the human specificity of the model, and other problems need to be solved urgently [11,131]. In addition, the translation of organoid models into clinical practice requires animal model validation to ensure the effectiveness and safety of organoid models. However, there are differences between animal models and humans, and the results of animal models need to be interpreted with caution.

#### Technology integration

The integration of AI technology with multi-omics technology into a single platform can enable more efficient and accurate data analysis and prediction. However, there are differences between AI and multi-omics techniques, and new algorithms and tools need to be developed for effective integration [123]; AI model training and prediction results are affected by many factors, such as data collection, algorithm, parameter settings, and so on, and this leads to the poor repeatability of the AI model. At the same time, the development and application of AI models also require a large amount of data, but the data sharing and collaboration mechanism is not perfect, which limits the development and application of AI models. Data sharing platforms and collaboration mechanisms are needed to facilitate data sharing and AI model development [132].

#### Clinical validation and translation

Clinical research is an important part to evaluate the effectiveness and safety of organoid models [52]. However, the design and implementation of clinical research should strictly follow ethical norms and clinical trial standards to ensure the reliability and reproducibility of research results. Clinical trials require a lot of time and capital investment, and organoid models also require regulatory approval to be used in clinical practice. However, the approval standards and processes of regulatory agencies for organoid models are not clear, which limits the clinical translation of organoid models.

#### Cost and accessibility

The establishment and application of AI and organoid models require expensive equipment and reagents as well as professional technology and knowledge, which limits the popularization and application of organoid models [133].

#### Ethical issues of organoids and AI

Organoid models involve human tissues and cells, and ethical issues need to be considered, such as informed consent, privacy protection, and data security. It is necessary to establish sound ethical norms and regulatory mechanisms to ensure that the application of organoid models conforms to ethical principles.

The training of AI models requires a large amount of patient data, which raises the issue of data privacy and protection. Alternatively, AI models may be subject to biases in the training data, leading to algorithmic bias and discrimination. Moreover, the prediction results of AI models often lack transparency and interpretability, which limits users’ trust in AI models. The wrong prediction or decision of AI models may also lead to serious consequences. It is necessary to clarify the responsibility and accountability of AI models and formulate the corresponding responsibility allocation mechanism.

### Possible resolution strategies

#### Data analysis and interpretation

Establish uniform standards for data collection, processing, and analysis, such as the use of standardized operating procedures, data sharing platforms, and best practices for data analysis. Rigorous data quality control processes, such as data cleaning, denoising, and bias correction, were implemented to ensure data quality and reproducibility [11]. Develop multi-omics data integration platforms, such as integrating genomics, transcriptomics, proteomics, and metabolomics data into one platform to provide more comprehensive tumor features and predictive models.

#### Model standardization and complexity

Develop and promote standardized organoid culture methods, such as optimizing medium components, culture conditions, and cell seeding density, to ensure organoid stability and reproducibility. A multicell coculture system, including tumor cells, immune cells, vascular endothelial cells, and stromal cells, was established to more fully mimic the tumor microenvironment [134]. More complex organoid models are constructed using organ-on-a-chip technology, such as combining organoids with microfluidic chips to simulate the hemodynamics and material exchange of the tumor microenvironment [135]. Researchers can use microfluidic platforms to dynamically culture organoids and screen drugs, so as to evaluate the efficacy and safety of drugs more effectively [12]. Biodegradable and biocompatible biomaterials, such as hydrogels and cellulose nanofibers, are developed as culture substrates for organoids to improve their stability and replicability [136,137]. 3D printing technology can accurately print cells and biological materials into 3D structures, so as to simulate the morphology and structure of tumors more accurately. For example, researchers can use 3D printing to construct organoids containing both tumor and normal cells, thus more realistically simulating tumor heterogeneity and microenvironment [12]. Interdisciplinary cooperation is a key driving factor to promote the standardization and complexity of PDO training models. For example, bioengineers and oncologists need to collaborate to develop new 3D-printed materials and microfluidic chips, while computer scientists can use ML techniques to make predictions about the drug response of organoids. This interdisciplinary collaboration will help drive advances in PDO culture technology and provide more powerful tools for cancer research and treatment.

#### Technology integration

Develop platforms that integrate AI and multi-omics technologies, such as image analysis, mutation prediction, and drug response prediction using AI technology, and combine multi-omics data to improve the accuracy and reliability of prediction. Establish bioinformatics platforms, such as gene expression databases, protein interaction networks, and drug response databases, to support organoid modeling and data analysis. Promote interdisciplinary collaboration between biologists, clinicians, computer scientists, and engineers to jointly address the challenges of organoid translation into clinical practice.

#### Clinical validation and translation

To improve clinical validation and translation, rigorously designed clinical trials can be used to explore more multidimensional genetic variation data and pathways to develop more accurate treatment plans. For example, factors such as tumor microenvironment, epigenetics, and multitarget combined treatment strategies are considered. Dynamic changes in tumor characteristics and under therapeutic pressure, such as target clearance and neuroendocrine transformation, are considered to guide more effective therapeutic strategies. AI and ML algorithms are used for data mining and analysis to optimize clinical trial design and improve the efficiency of patient recruitment and management [138]. Ding et al. [139] used droplet emulsion microfluidics technology to rapidly generate patient-derived micro-organospheres (MOS), which can evaluate tumor drug response within 14 days. Both time periods and clinical consistency were shown to guide clinical decision-making. MOS preserves the original tumor stroma and immune cells and is suitable for immuno-oncology therapy testing. This technique is high-throughput and time-sensitive and has shown a correlation with clinical outcomes in a clinical study of patients with metastatic CRC.

#### Cost and accessibility

Organoid culture techniques and data analysis methods need to be optimized to reduce costs and improve efficiency. Develop cost-effective AI solutions that reduce the need for computing power and data. Algorithms are optimized for efficiency and edge computing is explored to enable AI tasks to be executed on local devices. Establish a data sharing platform and collaboration mechanism to reduce the development, application, and computational cost of AI models, promote data sharing and AI model development, and improve their application in resource-poor settings.

#### Ethical issues of organoids and AI

The use of tumor organoids in the context of precision medicine involves multiple ethical considerations. Informed consent is the key. Patients need to fully understand the purpose, process, and potential risks of organoid culture to ensure that they can make their own decisions about participation. Second, privacy protection is crucial, and patient data should be kept strictly confidential to prevent leakage. The issue of equity cannot be ignored. We should ensure that all patients have equal access to the precise treatment brought by organoid technology. Finally, it is necessary to consider the rationality of resource allocation to avoid excessive concentration of resources that will affect other medical needs. The ownership of organoids also needs to be clarified. Organoids grown from a patient’s tumor tissue should remain the property of the patient, and any commercial use requires explicit authorization from the patient.

Ensure diversity and representation of AI training data, and regularly check and correct biases in AI models. The fairness evaluation index is introduced to ensure that the algorithm is fair and nondiscriminatory for all users. Strengthen data encryption and anonymization to ensure that user privacy is not violated, and implement transparent data collection policies that ensure users are clearly aware of how their data are collected, used, and stored, and that they are given the right to choose. Ensure that the decision-making process of AI systems can be explained, improve the transparency of algorithms, and encourage AI developers to provide detailed algorithm descriptions and decision-making processes, so that users can understand the decision-making basis of AI. An external audit mechanism is introduced to review and evaluate AI algorithms through independent third-party institutions to ensure their transparency and fairness. In addition, it is necessary to clarify the responsibility of AI when it makes mistakes and establish the corresponding legal and ethical framework.

## Conclusion and Prospect

**Fig. 6.**
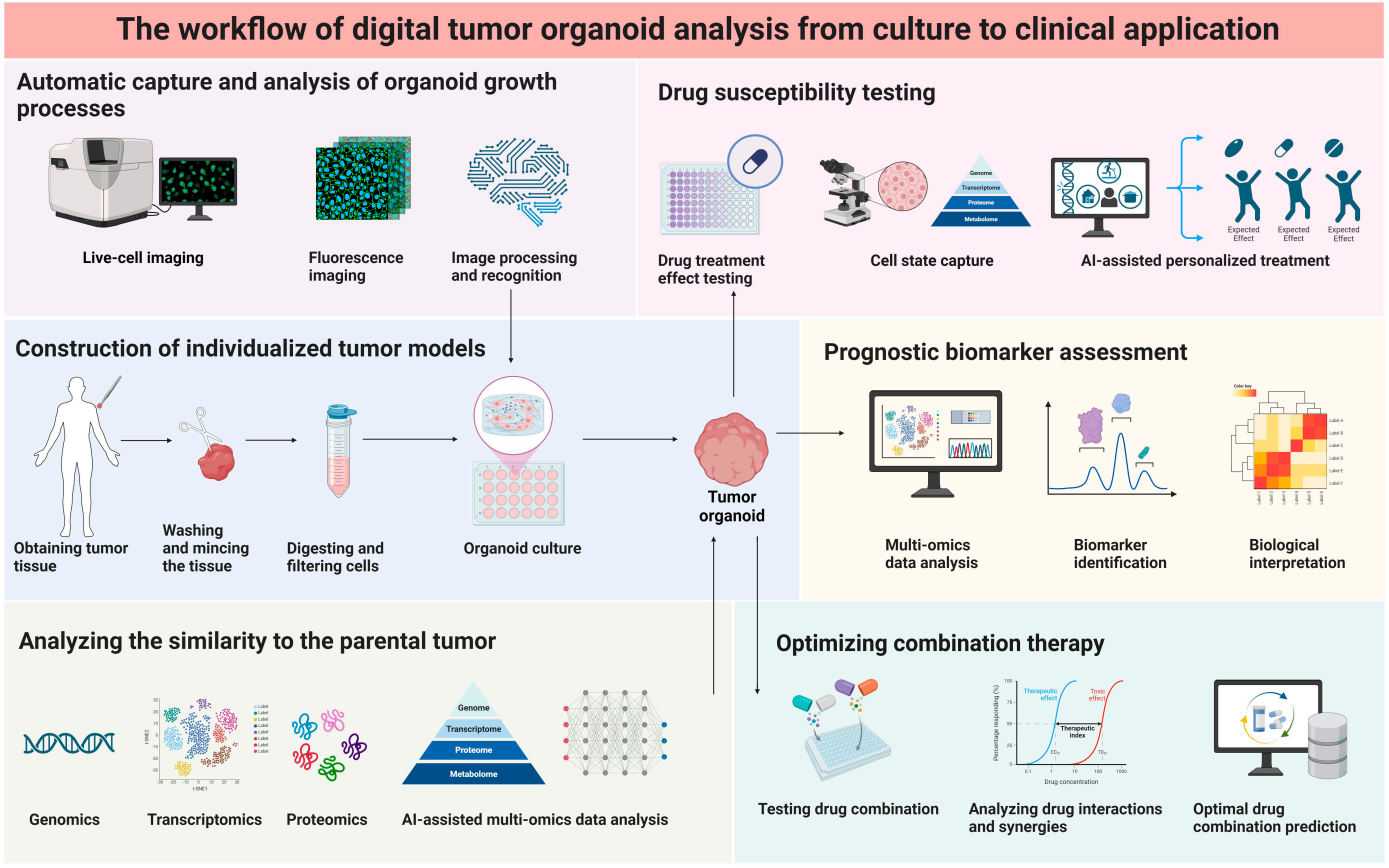
The workflow of digital tumor organoid analysis from culture to clinical application.

Tumor organoids are highly representative and reproducible 3D models that can simulate real tumor tissues. Through digital analysis methods, tumor organoids can be comprehensively and qualitatively studied at multiple levels, including genomics, proteomics, and metabolomics. These digital data can reveal the biological characteristics and heterogeneity of tumors and predict the response of tumors to drugs and drug resistance mechanisms, providing an important basis for the development of individualized treatment strategies. The collaboration of PDO with multi-omics or AI empowers higher similarity to the patient’s tumor, a deeper understanding of tumor heterogeneity, and more accurate prediction of tumor response and drug resistance; such models provide an important tool for precision medicine. In terms of precise diagnosis, biomarkers closely related to tumorigenesis and development can be discovered through genomic and proteomic analyses of tumor organoids of patient origin, providing a new basis for early diagnosis and prognosis assessment. In terms of precision therapy, tumor organoids can be used to test the efficacy of different drugs against tumor cells to identify the most effective treatment. It can also accelerate the development of new drugs and reduce the risk of clinical trials [140] (Fig. 6).

In conclusion, the digital analysis method for tumor organoids has important potential applications and development prospects for precision medicine. The highly automated culture and monitoring system will use sensors, microfluidic technology, and ML algorithms to realize real-time monitoring and automatic regulation of organoid growth to ensure its stability and consistency. The multidimensional data integration and analysis platform will integrate multiple data such as genome and transcriptome and reveal the biological mechanism of organoids through advanced analysis technologies such as deep learning, which will provide strong support for disease research and drug development. The establishment of virtual organoids will be based on real data and show the growth and reaction process of organoids through computer simulation, which will provide an efficient and low-cost alternative for experimental design and drug screening. The AI-driven optimization process will use AI algorithms to predict and optimize the culture conditions and drug reactions of organoids, and further improve their simulation and application value. Ultimately, combined with patient genomic data and organoid models, digital technology will enable the development of precision medicine and personalized treatment plans to select the most effective treatment drugs and doses for patients. Optimizing the technology, strengthening multidisciplinary cross-cooperation, and focusing on clinical application and translational research will promote the development and application of tumor organoids in precision medicine and enhance therapeutic effects and quality of life for patients with cancer.

## Supplementary Materials

Supplementary 1 Table S1 Reference [141]
