# Supplementary material for: A New Perspective on Precision Medicine: The Power of Digital Organoids
Source: Biomater Res. 2025 Mar 24;29:0171. doi: 10.34133/bmr.0171 (PMC11931648; doi:10.34133/bmr.0171)
Supplement: Supplementary 1 — Table S1 Reference [141] [file bmr.0171.f1.docx]

**Table S1.** Comparison of 2D cell lines, PDXs, and PDOs

| Feature | 2D cell lines | PDXs | PDOs |
| --- | --- | --- | --- |
| Expansion | +++ | + | ++[31] |
| Preserve histological characteristics of parental tumors | - | +++ | ++[44] |
| Preserve genetic characteristics of parental tumors | + | ++ | ++[45] |
| Retention of heterogeneity | - | ++ | ++[46] |
| Gene editing | +++ | - | +++[47] |
| Tumor modelling | ++ | - | +++[47] |
| High-throughput analysis | +++ | - | +++[48] |
| Drug sensitivity | +++ | ++ | ++[49] |
| Predict treatment response | + | ++ | ++[50] |
| Mimic tumor microenvironment | - | ++ | +[51] |
| Biobank | - | - | +++[46] |
| Cost | Cheap | Expensive | Medium[52] |

Note: +++, best; ++, good; +, possible; -, unsuitable. 2D, two-dimensional; PDXs, patient-derived tumor xenografts; PDOs, patient-derived tumor organoids.
